# Red light violation and pedestrians’ modal salient beliefs about unsafe road crossing behavior: a qualitative study

**DOI:** 10.5249/jivr.v11i2.1184

**Published:** 2019-07

**Authors:** Mahdi Moshki, Khajavi Abdoljavad, Doshmangir Leila, Saeid Pour Doulati

**Affiliations:** ^*a*^Health Education and Health Promotion Department, School of Health; Social Development and Health Promotion Research Center, Gonabad University of Medical Sciences, Gonabad, Iran.; ^*b*^Community Medicine Department, School of Medicine, Gonabad University of Medical Sciences, Gonabad, Iran.; ^*c*^Department of Health Services Management, School of Management and Medical Informatics, Tabriz University of Medical Sciences, Tabriz, Iran.; ^*d*^Health Services Management Research Center, Tabriz University of Medical Sciences, Tabriz, Iran.; ^*e*^Social Development & Health Promotion Research Center, Gonabad University of Medical Sciences, Gonabad, Iran.; ^*f*^Road Traffic Injury Research Center, Tabriz University of Medical Sciences, Tabriz, Iran.

**Keywords:** Attitude, Theory of Planned Behavior, Red light-Violation, Pedestrians

## Abstract

**Background:**

Pedestrians are amongst the most vulnerable road users and their unsafe behaviors have a major impact on traffic injuries. The aim of this study was to determine the underlying psychological factors behind red light violation in pedestrians’ crossing behavior based on the Theory of Planned Behavior (TPB) and to provide recommendations for preventive interventions.

**Methods:**

This qualitative study was conducted in Tabriz, one of the metropolitan cities of Iran. 30 pedestrians were individually interviewed using semi-structured, open-ended questions to elicit salient consequences, social referents, and circumstances regarding pedestrians’ red light crossing behavior. The transcribed interviews were analyzed using directed content analysis followed by frequency analysis in order to detect modal salient beliefs.

**Results:**

A total number of 115 sub-categories were identified which were then classified in the ten predetermined categories of the Theory of Planned Behavior: advantages, disadvantages, positive feelings, negative feelings, approving referents, disapproving referents, behaving referents, not-behaving referents, facilitators, and barriers. “Saving time” was elicited as the most important both the advantage and the positive feeling. “Getting injured” was identified as the most serious disadvantage. “Lowering the level of culture” was obtained as the main negative feeling. “Friends/Peers” comprise the most prominent group among both the approving and the behaving referents, whereas “Family members” constituted the most significant group both among the disapproving and the not-behaving referents. “Being in a hurry” was introduced as the most substantial facilitator and “The fear of accident” was identified as the most influential deterrent factor.

**Conclusions:**

Based on the major findings, reducing pedestrians’ red light violations needs to focus: on the perceived negative and positive consequences and feelings of this behavior like getting injured, and saving time respectively; on the approval role of friends/peers, and the disapproval role of family; and on the fear of accident as a barrier, and rushing as a facilitating factor. More precise quantitative research is needed to determine the predictive power of these factors in such risky behavior.

## Introduction

Pedestrian injuries and fatalities have emerged as an increasing cause of concern worldwide.^1^ 90% of global traffic injuries occur in low-middle income countries where vulnerable road users with less protection including pedestrians comprise nearly half of the traffic injury victims.^2,3^ Pedestrians are more at risk in urban areas, where 55-75% of the traffic mortality is accounted for by pedestrians.^4^ To protect pedestrians and other vulnerable road users, the World Health Organization (WHO) recommended governments to translate effective, evidence-based knowledge into policies and practices.^5^ Despite the high injury and mortality rate of pedestrians and the importance of the human factor, which emphasizes the implementation of evidence-based safety promotion programs, insufficient attention has been paid to pedestrian safety.^3,6-10^

Pedestrian injuries occur for many reasons, especially unsafe behavior and failure to comply with traffic rules.^11-13^ One of the most common forms of risky pedestrian behavior is crossing the road against a red light.^13^ In Iran, half of the traffic accident victims are pedestrians and motorcycle users.^10^ 38% of pedestrian accidents in Tehran (the capital city ) are related to the risky and unsafe pedestrian behaviors.^14^ In order to improve these unsafe behaviors one needs to fully understand the individual-level underlying psychological factors, influencing pedestrians’ traffic behavior.^15^ The theory-based qualitative data can provide the information needed to understand these factors and develop targeted interventions that are more effective than non-theoretical interventions.^16-18^ The Theory of Planned Behavior (TPB) is one of the most validated theories of individual-level behavior that has been used to predict pedestrians’ behavior.^19-26^

The TPB states that behavioral, normative and control beliefs through attitudes, subjective norms and perceived behavioral control predict intention and behavior.^15,16,27-29^ Investigating attitudinal, normative and control beliefs are the basis for the development of interventions.^30,31^ The salient beliefs are the limited number of many beliefs an individual has about a certain behavior at any given time. These beliefs emerge through formative research by conducting a qualitative survey in a group of representative participants from the target population.^30,32^ Although few quantitative studies suggest that TPB can accurately predict pedestrians’ intention, qualitative studies for a better understanding of underlying cultural factors are a matter of great importance. Investigation in the field of TPB revealed that only a limited number of researchers have used formative qualitative studies to identify targeted interventions.^33^ To the best of our knowledge, no TPB-based qualitative study has ever been conducted to understand pedestrians' road crossing intention or behavior.^19,21-26,34^

We applied TPB as a theoretical framework to explore the underlying psychological factors predicting Pedestrians’ Red Light Violation Behavior (PRLVB) in Tabriz. The metropolis of Tabriz is the capital of East Azarbaijan province, located in the northwest of Iran, and has about 1.5 million inhabitants. Applying this theory to elicit pedestrians’ beliefs can be used to design effective interventions to improve their risky behavior and also act as a source of information in the development of a TPB-based questionnaire. 

## Methods 

In this qualitative study, based on the interpretivist paradigm, we used individual interviews to obtain pedestrians’ salient beliefs through directed content analysis followed by frequency analysis. The target behavior was: crossing the road at an intersection, on a normal day while the traffic light is red for the pedestrian. The study period was from May 2018 to Sep 2018.

***Participant selection ***

We followed the Jillian Francis method to determine the number of participants in a theory-based qualitative study.^35^ It argues that researchers need to determine in advance how many primary participants are required to get interviews started. Then, they should fix the stopping point. To conclude that data saturation has been achieved, they have to determine how many more successive interviews are required if a new theme is not extracted. So, considering the research question, the number of likely dimensions of the constructs, and the diversity of participants (in terms of age, sex, education, occupation, marital status, and signalized intersection crossing status) we set the initial number of participants at 18. Then we continued with three successive interviews. Since in the three consecutive interviews of participants 28, 29, and 30, no new codes developed, we reached data saturation. A summary of participants’ characteristics is presented in Table 1.

**Table 1:** Participants’ characteristics.

Age (N=30)		Sex (N=30)		Education (N=30)		Occupation (N=30)		Marriage (N=30)		Crossing (N=30)	
**Mean**	40.93	Male	15	No diploma	1	public employee	14	married	21	Cross	15
**Median**	39.00	Female	15	Diploma	6	private employee	2	single	9	Not cross	15
**SD**	13.06			Associate degree	1	self-employee	6				
**Min**	21			Bachelor degree	18	retired	4				
**Max**	75			Master degree	3	students	2				
****				Doctorate	1	house worker	1				

***Data collection instrument and data collection method***

We used a semi-structured, theory-based questionnaire^36,37^ for the interview (Table 2). Initially, we introduced a scenario as follows: suppose a pedestrian arrives at a signalized intersection, and crosses the road while the traffic light is red for pedestrians. Then we started the interview asking 13 questions in total which had been guided by the independent variables of the TPB. Questions on advantages/disadvantages were designed based on the Ajzen & Fishbein recommendation^36^ and questions on positive/negative feelings based on the Ajzen & Driver.^37^ To reduce the response bias, the order of experiential and instrumental questions was balanced. So that half of the participants first responded to the instrumental questions, then to the experiential questions, and vice versa.^38^

The inclusion criteria were: age over 17, individuals who are crossing or intended to cross at a signalized intersection, lack of physical and mental disabilities, and lack of severe mental disorder. We chose the age as over 17 so that the participants could provide a deep understanding of the pedestrians’ red light crossing behavior. As recommended by Fishbein & Ajzen,^30^ we selected the three intersections, where pedestrians are somehow representative of the whole city population and there is a maximum degree of homogeneity within the group. To reach the minimum primary study participants of 18, six people at each intersection were randomly selected. A team of interviewers, consisting of a man and a woman at various hours of the day, approached the pedestrians, introduced themselves, explained the objectives of the study, and checked them for the inclusion criteria. Eligible people, after signing the informed consent form, were invited to the nearest health center for interview, or any place they want. Half of the participants were men and the other half were women, half intended to cross on the red light, and the other half did not. Each interview lasted 40 to 60 minutes, and the answers were written down word-by-word.

**Table 2:** Open-ended questions used for belief elicitation.

No	QUESTIONS
**1**	What do you see as the advantages of crossing when a pedestrian red light is showing?
**2**	What do you see as the disadvantages of crossing when a pedestrian red light is showing?
**3**	What do you like about pedestrians’ red light crossing behavior?
**4**	What do you dislike about the pedestrians’ red light crossing behavior?
**5**	What else comes to mind when you think about the pedestrian red light crossing behavior?
**6**	Please list the individuals or groups who would approve or think you should cross on a pedestrian red light.
**7**	Please list the individuals or groups who would disapprove or think you should not cross on a pedestrian red light.
****	Sometimes, when we are not sure what to do, we look to see what others are doing.
**8**	Please list the individuals or groups who most likely to cross when a pedestrian red light is showing.
**9**	Please list the individuals or groups who least likely to cross when a pedestrian red light is showing
**10**	What else comes to mind when you think about the social referents’ viewpoints/directions about the pedestrian behavior when a red light is showing?
**11**	Please list any factors or circumstances that would make it easy or enable you to cross when a pedestrian red light is showing.
**12**	Please list any factors or circumstances that would make it difficult or prevent you from crossing when a pedestrian red light is showing
**13**	What else comes to mind when you think about the facilitating or hardening factors or circumstances related to pedestrian red light crossing behavior?

***Data analysis***

TPB-based qualitative studies usually use a directed content analysis approach, so that the theoretical framework guides the analysis rather than the data.^32,38^ In the first step (overview), the principal researcher along with the other two researchers independently read and reread the answers to get insight into the participants' beliefs. In the second step (structuring and generating), the first six responses were used to create the coding framework by the principal researcher, and this was later employed to encode the remaining responses by the other two researchers. ^32,38^ In this step, he constructed the main categories and created the subcategories. The content-driven way was used to construct the main categories and the data-driven way to generate subcategories which are mutually exclusive within the main category. Using the subsumption method in the data-driven way, he generated sub-categories.^32,38^ This was done in such a way that, the responses were read and immediately used for the generating of meaning units. Each of these meaning units represents one of the main categories.^39,40^ The meaning units that were close together were merged into the condensed meaning units called sub-categories.^41^In the case of a new concept, if it was possible to categorize it within the previous sub-categories, it would be allocated to one of them, otherwise, it would be used to generate a new sub-category.^38^ We used the same coding frameworks for the advantages and the positive feelings questions; the disadvantages and the negative feelings questions; approving referents, disapproving referents, behaving referents, and not behaving referents questions. But for the questions on facilitators and for the questions on inhibitors, a different coding framework was used. ^42^ The process of generating sub-categories is frequently repeated as suggested by Condole. ^43^

Then, two researchers independently analyzed the remaining 24 interviews based on the coding framework obtained from the previous stage. At this stage, in the event of being faced with a new concept, if it was possible to allocate it to one of the previous sub-categories, we would do so; otherwise, it would be used to generate a new sub-category in consultation with the other coder and any disagreement would be resolved by the principal investigator. A sample of meaning units, condensed meaning units (sub-categories) and categories is provided in Appendix-Table A1. Then the number of sub-categories in each category was calculated separately (see Appendix- Table A2).

**Table A1:** Sample meaning units, condensed meaning units, sub-categories, and categories of the PRLVB

Meaning units	Condensed meaning units	Sub-categories	categories
• By doing this behavior, I get to the destination more quickly and so lose less time.	Time saving	Advantages	Attitude
• The only benefit of this behavior is that: it is fast and some people don’t want to wait.			
• Maybe saving a little time.			
• A person who crosses the pedestrian light causes chaos and disorder in the city.	Disrupting City System	Disadvantages	
• One of the biggest disadvantages of this behavior is disrupting the public discipline and city's appearance.			
• This behavior disrupts the rules and order of the community.			
• I like this behavior because I could get the destination more quickly.	Time saving	Positive feelings	
• I do not like to wait for green lights and I want to arrive soon.			
• Lowers my level of culture.	Lower the level of culture	Negative feelings	
• This behavior is contrary to the culture of urbanization.			
• I do not like to be an uncultured person.			

**Table A2:** The total number of meaning units, sub-categories, modal salient beliefs and the Cohen Kappa coefficient for each main categories

Main categories	Meaning units (N*)	Sub-categories (N*)	Modal salient beliefs (N*)
The advantages of PRLVB	28	4	1
The disadvantages of PRLVB	100	13	10
The positive feeling of PRLVB	11	5	1
The negative feeling of PRLVB	73	11	9
Approving referents of the PRLVB	23	7	4
Disproving referents of the PRLVB	86	14	8
Behaving referents of the PRLVB	38	11	7
Not behaving referents of the PRLVB	74	17	6
Facilitator factors of the PRLVB	67	20	7
Hindering factors of the PRLVB	61	13	7
**Total**	**561**	**115**	**60**

* N=Number

After generating sub-categories and assigning them to the relevant categories, in the next step, we proceeded with the use of frequency analysis to identify the modal salient beliefs. Ajzen and Fishbein suggested three rules to determine the most frequently mentioned (modal) beliefs: 

1. Take the 10 or 12 most frequently mentioned beliefs.

2. Take all the beliefs mentioned by at least 10% or 20% of the participants.

3. Take as many beliefs as needed to make up a certain percentage (eg. 75 %) of all responses listed.^44^

We chose the items that were mentioned by at least 10% of the participants as modal salient beliefs.

***Ethical consideration***

We provided the necessary information to the participants regarding the issues being investigated and the objectives of the study. They were also assured about the confidentiality of the information obtained, the right to choose the time and place of the interview and the right to withdraw from the study at any time. Permission to record the interviews was obtained from the participants.

***Trustworthiness***

As an investigator triangulation we involved two independent coders to analyze the content of the interviews. By peer debriefing, reviewing research and data by someone outside the research group who is familiar with the research and pedestrian behavior, and member checking by the six participants, providing data analyses and results to the participants and taking their feedback, we assured trustworthiness.^45^ To increase the validity of the study, the codes were reviewed and approved by an injury prevention and pedestrian safety promotion specialist. Also, the researchers' notes, final report and general approach were reviewed by him, and then the necessary feedback was provided both verbally and in written form.^46^ Participants were asked to look at the raw data and comment on their accuracy. Also, they were asked to comment on the significance of the categories and sub-categories; if they were exclusive and real; and if they had been built on sufficient evidence. Participants' comments were considered in the final version.^46^

## Results

***Main categories and sub-categories***

Using the structuring method and content-driven approach, ten main categories based on the theory of planned behavior components were constructed: 1. Advantages, 2. Disadvantages, 3. Positive feelings, 4. Negative feelings, 5. Approving referents, 6. Disapproving referents, 7. Behaving referents, 8. Not behaving referents, 9. Facilitating factors, and 10. Hindering factors. We generated 4-20 sub-categories for each category by the data-driven approach (Appendix-Table A2).

***Salient beliefs***

Table A2 (see Appendix) lists the total number of meaning units, the number of condensed meaning units obtained from the directed content analysis, and the number of modal salient beliefs related to each of the ten main categories.

The lowest number of extracted meaning units is related to the negative feelings about PRLVB and the highest one goes to the disadvantages of PRLVB. The lowest number of generated sub-categories is related to the advantages of PRLVB and the highest one is linked to the facilitator factors of PRLVB. The lowest number of modal salient beliefs is related to the advantages and positive feelings and the highest one belongs to negative feelings. 

Table A3-A7 (see Appendix) highlight the pedestrians’ modal salient beliefs regarding the PRLVB based on the TPB components. These beliefs include modal salient instrumental and experiential consequences underlying attitude, modal salient injunctive and descriptive referents underlying perceived norms, and modal salient circumstances underlying perceived control.

**Table A3:** Modal salient instrumental consequences of the PRLVB

Advantages (n=30)	No. (%)		Disadvantages (n=30)	No. (%)
Time saving	25(83/3)		Getting injured	27(90)
			Disturbing City System	14(50)
			Breaking the Law	12(40)
			Financial Damage	10(33.3)
			Violating Citizenship Rights	10(33.3)
			Traffic Jam	7(23.3)
			Lower the Level of Culture	6(20)
			Bad Education in the society	4(13.3)
			Psychological Consequences	4(13.3)
			Creating Tension in the society	3(10)
	**25**			**94**

**Table A4:** Modal salient experiential consequences of the PRLVB

Positive feelings (n=30)	No. (%)		Negative feelings (n=30)	No. (%)
Time saving	7(23.3)		Lower the Level of Culture	15(50)
			Breaking the Law	15(50)
			Disturbing City System	12(40)
			Getting injured	8(26.7)
			Violating Citizenship Rights	5(16/7)
			Negative View Toward Offender	4(13/3)
			Psychological Consequences	4(13/3)
			Personality Weaknesses	4(13/3)
			Bad Education	3(10)
	**7**			**70**

**Table A5:** Modal salient injunctive referents of the PRLVB

Approving referents (n=30)	No. (%)		Disapproving referents (n=30)	No. (%)
Friends/Peers	8(27)		Family	22(73/3)
Relatives	6(20)		Police	16(53/3)
Family	3(10)		Educated People	13(43/3)
Colleagues/Classmates	3(10)		Friends/Peers	10(33/3)
			Colleagues/Classmates	7(23/3)
			Relatives	4(13/3)
			Mass Media	4(13/3)
			Law-abiding People	3(10)
	**20**			**79**

**Table A6:** Modal salient descriptive referents of the PRLVB

Behaving referents (n=30)	No. (%)		Not behaving referents (n=30)	No. (%)
Friends/Peers	8(26.7)		Family	20(66.7)
Relatives	7(23/3)		People Educated	12(40)
Colleagues/Classmates	4(13/3)		Friends/Peers	9(30)
Youth /Teens	4(13/3)		Police	9(30)
Family	3(10)		Colleagues/Classmates	8(26.7)
General public	3(10)		Relatives	5(16.7)
Stupendous People	3(10)			
	**32**			**63**

**Table A7:** Modal salient circumstances of the PRLVB

Facilitating factors (n=30)	No. (%)		Hindering factors (n=30)	No. (%)
Being in a hurry	17(56/7)		Fear of Accident	14(46/7)
Physical Ability	8(26/7)		The Police Presence	14(46/7)
Escape Rule	5(16/7)		Not Crossing other Pedestrians	9(30)
Absence of deterrent provisions	5(16/7)		Complying with the Law	7(23/3)
Calm street	5(16/7)		Deterrent Equipment such as fences, traffic signs and cameras	4(13/3)
Psychological problems	4(13/3)		Penalty for Offender	4(13/3)
Police permission	4(13/3)		Presences of a Child or a Disabled person	3(10/0)
	**48**			**55**

***Beliefs on salient consequences underlying instrumental attitudes and beliefs on salient feelings underlying experiential attitudes ***

The beliefs underlying instrumental attitudes towards PRLVB are positive or negative consequences of red light violation and the beliefs underlying experiential attitudes are positive and negative feelings of red light violation. Table A3 and A4 (see Appendix) illustrate the modal salient beliefs of the instrumental and experiential attitude toward the PRLVB. In both tables, the participants have expressed far more negative outcomes than positive ones. The analysis of the content of 28 responses to the advantage question and 100 responses to the disadvantage question revealed that 1 advantage and 10 disadvantages were reported by at least 10% of the participants. The most common perceived advantages and disadvantages were time-saving and getting injured respectively. Disturbing the city system, expressed by half of the participants, was the second modal salient belief from the disadvantages.

Out of five positive feelings and 11 negative feelings regarding the PRLVB, 1 and 9 were mentioned by at least 10% of the participants, respectively. Time-saving similar to the instrumental consequences was recognized as the most significant positive experiential consequence, the difference being that only 23.3 per cent of the participants would cross on a red light in order to save time. In contrast, the feeling of lowering the level of culture and breaking the law mentioned by 50% of the participants was the most perceived negative perception of this behavior.

***Beliefs on salient referents underlying injunctive and descriptive norms ***

Two types of social referents are involved in the perceived norm: the injunctive normative referents, who approve or disapprove of a given behavior, and descriptive normative referents, who do or do not behave in practice. Table A5 (see Appendix) presents injunctive normative referents. According to this table, the participants mentioned a relatively large number of disapproving referents compared with those approving this behavior (8 versus 4). Friends/peers, mentioned by 27% of the participants, constitute the highest frequency of approving referents and family members (73.3%) constitute the highest disapproving referents. This is while family members have been mentioned by just 10% of participants. Relatives play a role of approving referents more than family members. And colleagues/classmates with 10% have a similarly low frequency as family members as approving referents. In the disapproving group, the police (53.3%) and educated people (43.3%) are the most frequently mentioned group after family members. Friends/peers, which ranked first with 27% frequency as the approving referent, ranked fourth with 33.3% frequency within the disapproving category.

Table A6 (see Appendix) presents the modal salient descriptive normative referents. In the present study, these referents include individuals or groups who are most likely or least likely to cross the road when the light is red for pedestrians. According to this table, although the number of sub-categories in these two categories is very close to each other (7 versus 6), the frequency of behaving referents compared to the not-behaving referents is almost double (63 vs. 32). Friends/Peers represented by 26.7% of the participants have the highest frequency among the behaving referents and family members with the frequency of 66.7% have the highest frequency among not-behaving referents. The police are the group that is placed only on the category of the not-behaving referents, and the youth, teens and the general public are those that are placed only on the behaving group. Other groups including relatives, colleagues/classmates, family members, friends/peers, and educated people are common in both categories, albeit with a higher frequency in the not-behaving referents, with the exception of relatives which have a higher frequency in the behaving group (7 versus 5).

***Beliefs on salient circumstances underlying perceived control ***

Table A7 (see Appendix) presents the facilitating and hindering factors for PRLVB. Overall, the hindering conditions had more frequency than the facilitating conditions, but the number of modal ones is equal in both categories (7 versus 7). Being in a hurry introduced by 56.7% of participants was the most frequent enabler, and fear of accident which is introduced by 46.7% of the participants is the most common impeding factor. The second facilitator is the physical ability to cross the road quickly. Having features of escaping the law and the absence of deterrent provisions are considered as the next most important facilitating factors. The presence of the police (46.7%) is identified as the second leading cause of hindering factor. Other pedestrians who are not crossing (30%), complying with the law (23.3%) and the existence of deterrent equipment such as fences and cameras (13.3%) recognized as the other modal salient barrier.

## Discussion

Although several studies have been conducted based on the TPB to investigate pedestrians’ unsafe crossing behavior, none of them have conducted a qualitative study as a prerequisite for the quantitative study.^19-26^

To the best of our knowledge, this is the first qualitative research aimed to elicit pedestrians' modal salient beliefs regarding PRLVB. The results of this study could be used for designing pedestrian injury prevention interventions. Moreover, this qualitative research provides necessary information for the design of a quantitative questionnaire and validation of the theoretical structure of the TPB in predicting PRLVB. In fact, this study is an essential prerequisite for understanding pedestrians' unsafe crossing behavior, and will help injury prevention and safety promotion planners to focus on the beliefs that have the greatest impact on the pedestrians' unsafe crossing behavior. The results of this study are the salient consequences underlying instrumental and experiential attitudes; the salient referents underlying injunctive and descriptive norms; and the salient circumstances underlying perceived behavioral control. Since we could not find a similar qualitative study on the elicitation of pedestrians' modal salient beliefs regarding pedestrians' unsafe crossing behavior, we compared the results of this study with the quantitative studies that predict pedestrians’ unsafe crossing intention or behavior in China, UK, and Columbia based on the TPB. This comparison indicates that the results of the present study have some similarities and differences with the above-mentioned studies.

***PRLVB consequences***

The first finding of the present study is the pedestrians' beliefs about the advantages, disadvantages, positive feelings and negative feelings regarding PRLVB. According to the findings of the present study, the participants did not see many advantages from this behavior and time saving was the only positive consequence. Therefore, interventions that emphasize the pedestrians' minimum waiting time are of great importance. But, given that the average waiting time for pedestrians in each signalized intersection is usually less than one minute, the value of this time saving, as compared to the disadvantages of this behavior, especially getting injured, should be emphasized in the educational programs.^47^ At the same time, as highlighted in the previous studies, authorities should also focus on community-based systems to meet the psychological needs of pedestrians, such as reducing waiting time.^6^ Time-saving as an attitudinal question has been used in several studies in China, UK and Colombia.^19-25^ In three studies conducted in UK and China,^20,21,25^ researchers in addition to the time-saving, have used another item like get me to my destination more quickly, as one of the advantages of this behavior. But we combined these two items because of the overlap in their meanings and put them in a single sub-category called time savings. 

Contrary to the advantages, participants indicated many disadvantages from this behavior, of which getting injured has more frequency. This disadvantage was considered as an attitude item in five studies conducted in China, UK, and Colombia,^19,20,22,23,25^ although, in two other studies conducted in China^24^ and UK,^21^ it did not exist. Among the other disadvantages, disturbing the city system which was declared as the second disadvantage by half of the participants was not included in the previous studies. The specific socio-cultural status of each society, the lack of a qualitative study before designing a quantitative questionnaire, and the changes of the items which could occur during the process of validity and reliability could be reasons for these differences. The findings of this study indicate that in total, participants did not have a positive attitude towards this behavior insofar as, on average, less than half a belief per person is expressed for this question. However, as mentioned for the advantages, time-saving is known as the only salient cause of having a positive attitude towards this behavior as well, but this view is only quoted by a quarter of the people. However, participants through different perspectives, including lowering the level of culture and breaking the law, that each of which has been described by half of the participants, had a negative feeling to the PRLVB.

***PRLVB social referents***

The second finding of this study is related to the pedestrians’ quadruple salient referents, who are approving, disapproving, behaving, and not behaving referents. The number of disapproving referents is considerably more than approving referents. The higher number of disapproving referents reflects the inappropriateness of this behavior as a social norm. Friends/peers were the most important referents who approve, and family members were the most important referents who do not approve of the PRLVB.

Therefore, for the effectiveness of the educational and interventional programs, more attention should be paid to the role of friends/peers as the approving referents and the role of the family members as the disapproving referents in preventing such risky behavior. Family members, relatives, and colleagues/classmates are the other approving referents of this behavior that are ranked below, are also included in the list of disapproving referents. While the police, educated people, mass media and law-abiding people are those who only appear on the list of disapproving referents. In fact, these last four groups are not considered to cross on the pedestrian red light at all. Identifying and differentiation of approving and disapproving referents as a targeted group for intervention planning are of utmost importance to policy-makers and injury prevention planners.

According to the participants, the number of people or groups who most likely to cross on the pedestrian red light is considerably less than those who are unlikely to cross. So that, the most important referents who most likely to cross the pedestrians red light are the friends/peers, whom also recognized as the most important social referents approving this behavior. The most important not behaving referents are family members, who are at the same time regarded as the most important disapproving referents.

This issue doubles the importance of the two groups of friends/peers and family members being among the targeted educational and interventional groups. Individuals and groups that have been used as social referents in previous surveys include family members and friends in China,^24^ police, parents, drivers and other pedestrians in China^22^ friends, colleagues/classmates and adjacent pedestrians in China;^23^ other pedestrians; drivers and public figures in Colombia;^19^ friends; other pedestrians; In UK,^21^ family , friends , police and drivers in China;^26^ and friends and other pedestrians in the UK.^20^ Of the seven studies mentioned above, friends were the 5 most commonly used as social referents.

***PRLVB control factors***

The third finding of this study is the salient beliefs on facilitating and hindering factors underlying perceived behavior control. Being in a hurry is the most important factor convincing pedestrians to cross the road even if, the light is red for them. This factor is consistent with the time-saving factor, which is recognized as the main advantage of this behavior. This emphasizes the need for the culturalization of traffic regulations, including in a hurry. This should be synchronous with the provision of the necessary infrastructure for the pedestrians’ safety, including pedestrian traffic lights that are activated by pedestrians. Previous studies indicated that the provision of infrastructure, the strict implementation of traffic rules, and training of the traffic principles for pedestrians and drivers could significantly improve pedestrian safety in countries like Germany and the Netherlands.^48^ The physical inability to cross the road while the light is red for the pedestrians and the vehicles are travelling is the second factor that can facilitate this behavior. In terms of the deterrent factors, fear of collision is the first and the presence of the police is the second most important factor that could prevent pedestrians from red light crossing. Of course, if the necessary resources are provided, along with supplying other necessary infrastructure and in the presence of intelligent transportation systems, it can be effective to reduce pedestrian injuries. ^49^

Although this study uses a qualitative approach to elicit pedestrians’ beliefs about PRLVB, it suffers from some limitations. Similar to other qualitative studies the findings of this study have a poor generalizability. In order to assure representativeness of the participants and comprehensiveness of the obtained information, we tried to maximize diversity in the variables such as sex, urban areas and behavior (intention to cross vs. not cross) but for each community depending on the cultural characteristics of that community, it should be studied separately. There were no codes of sub-categories that do not fit the pre-defined categories based on the TPB. But if there was, a new category could be created for it. 

## Conclusion

This study elucidated the belief structure of the PRLVB. Friends/peers were shown as the most important approving and behaving group and family members as the most important disapproving and not-behaving group of pedestrians’ red light crossing behavior could be considered as target groups for educational interventions to modify pedestrians’ unsafe road crossing behavior. Considering the relatively positive attitude toward the pedestrian red light crossing behavior due to the saving time in one hand and being in a hurry as a facilitator of this behavior on the other hand, demonstrating the importance of the disadvantages or negative feelings of this behavior like getting injured, would be effective in preventing pedestrians’ red light violation. Of course, the items obtained from this qualitative research should be more accurately investigated by quantitative research to determine the predictive power of TPB constructs for the pedestrians unsafe crossing behavior and to investigate the impact of the TPB-based interventions on improving pedestrians crossing behavior.
